# Development and Content Validity of a Pre-participation Evaluation Tool for Recreational Athletes Engaged in High-Intensity Sports

**DOI:** 10.7759/cureus.103197

**Published:** 2026-02-08

**Authors:** Smita C Patil, Anandh S.

**Affiliations:** 1 Sports Physiotherapy, Krishna Vishwa Vidyapeeth (Deemed to Be University) Karad, Karad, IND; 2 Community Health Physiotherapy, Krishna Vishwa Vidyapeeth (Deemed to Be University) Karad, Karad, IND

**Keywords:** content validity, face validity, high-intensity sports, lawshe cvr, pre-participation evaluation, recreational athletes

## Abstract

Background: Participation in high-intensity sports among recreational athletes has increased substantially, accompanied by a higher incidence of preventable cardiovascular and musculoskeletal adverse events. Existing pre-participation evaluation (PPE) tools are largely designed for elite or scholastic athletes and frequently lack rigorous psychometric validation for recreational populations.

Objective: To develop a novel PPE questionnaire tailored for recreational athletes engaged in high-intensity sports and to establish its face validity and content validity using a systematic, theory-driven methodology.

Methods: Instrument development followed a two-phase qualitative design. Phase I involved a Preferred Reporting Items for Systematic Reviews and Meta-Analyses (PRISMA)-guided systematic review and expert consultation to define relevant domains and generate an initial item pool. Phase II focused on tool development and validation, including face validity assessment by a multidisciplinary panel of 20 experts and content validity evaluation using Lawshe’s content validity ratio (CVR). Pilot testing was conducted on 30 recreational athletes to assess feasibility and clarity.

Results: The systematic review and expert consultation yielded an initial pool of 65 items across five domains. Following face validity assessment, several items were revised or eliminated for clarity and relevance. Content validity analysis using CVR resulted in retention of 59 items, with 80% of items achieving CVR values ≥0.49. Pilot testing demonstrated high acceptability, clarity, and feasibility, with the majority of participants reporting ease of administration and reasonable completion time.

Conclusion: The developed PPE instrument demonstrates excellent face validity and strong content validity for recreational athletes participating in high-intensity sports. The final 59-item questionnaire provides a comprehensive, context-specific screening tool and is suitable for progression to reliability testing and further psychometric evaluation in larger prospective cohorts.

## Introduction

Recreational participation in high-intensity sports, including endurance running, competitive fitness training, high-intensity interval training, and amateur competitive sports, has increased globally [1,2]. While such participation confers substantial health benefits, it also elevates the risk of acute cardiovascular events, overuse injuries, and sudden musculoskeletal trauma, particularly among inadequately screened individuals [3-5].

Pre-participation evaluation (PPE) aims to identify health conditions and risk factors that may predispose athletes to adverse events during sport participation [6]. However, most PPE frameworks are derived from elite, youth, or intercollegiate athlete populations [7,8]. Recreational athletes differ significantly in age distribution, training supervision, medical oversight, and health heterogeneity, necessitating population-specific screening tools [9].

A critical limitation of existing PPE instruments is the absence of rigorous psychometric validation, particularly with respect to content validity, which is foundational to all subsequent measurement properties [10,11]. According to modern measurement theory, a tool cannot be considered valid if it fails to adequately represent the construct it intends to measure [12].

For the purpose of this study, recreational athletes were defined as individuals participating in organized or unorganized sports activities without professional or elite-level competition status. High-intensity sports were operationally defined as activities involving sustained or intermittent exertion ≥6 metabolic equivalents (METs), high cardiovascular demand, and/or rapid neuromuscular actions such as sprinting, jumping, cutting, or repeated high-load movements. Examples include football, basketball, competitive badminton, tennis, endurance running, and structured high-intensity training programs.

Therefore, the purpose of this study was to develop a novel PPE instrument specifically for recreational athletes engaged in high-intensity sports and to establish its face validity and content validity using best-practice methodological standards [11,13]. The present study was conducted with an objectives of identifying key domains relevant to PPE in recreational athletes engaged in high-intensity sports through a systematic literature review and expert consultation, developing a draft PPE questionnaire based on identified domains and expert consensus, establishing face validity and content validity of the PPE tool using structured expert evaluation methods and assessing feasibility and clarity of the tool through pilot testing in recreational athletes.

## Materials and methods

Ethical approval for this study was obtained from the Institutional Ethics Committee of Krishna Institute of Medical Sciences Deemed to be University (KVVDU), Karad. All expert panel members and recreational athlete participants provided written informed consent prior to participation. The study was conducted in accordance with the ethical principles outlined in the Declaration of Helsinki.

Study design and setting

This methodological study was conducted at Krishna College of Physiotherapy, KVVDU, Karad. The study was carried out in two major phases: Phase I: Systematic review and item generation, and Phase II: Development of the PPE tool and validity assessment.

The institution provided the necessary academic, clinical, and professional environment to support tool development and validation involving both experts and recreational athletes. Phase I focused on domain identification and item generation, using inputs from a systematic review and expert consultation, which resulted in a 65-item draft tool. Phase II involved validation and refinement of the draft instrument through face validity assessment, calculation of the content validity ratio (CVR), and the item-level content validity index (I-CVI). Items were retained based on predefined criteria (CVR ≥ 0.49 and I-CVI ≥ 0.78), leading to a refined 59-item tool.

Phase I: systematic review and item generation (qualitative study design)

Aim

Phase I aimed to identify relevant domains for a PPE tool specific to recreational athletes engaged in high-intensity sports and to generate an initial pool of items grounded in scientific evidence and expert opinion.

Participants (Experts)

A purposive sample of experts was recruited, including orthopedicians, physicians, physiotherapists, and sports medicine specialists with substantial clinical experience in athlete assessment and management. The primary inclusion criterion was willingness to actively contribute professional input. The multidisciplinary composition ensured that medical, musculoskeletal, and functional aspects of athlete readiness were comprehensively addressed. Expert involvement in tool development is widely recognized as essential for achieving content credibility and clinical usability [14,15].

Procedure

**Figure 1:**
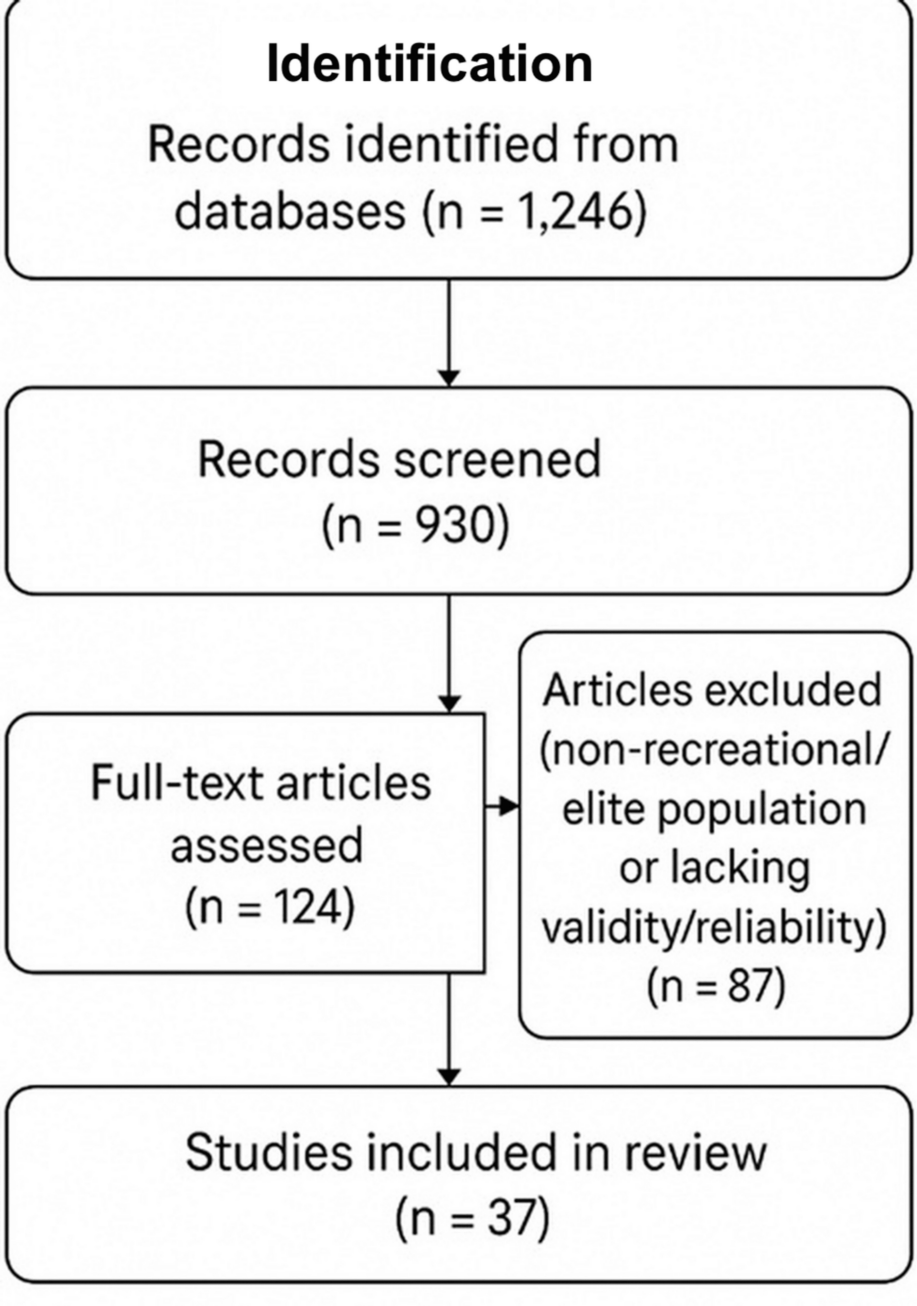
Preferred Reporting Items for Systematic Reviews and Meta-Analyses (PRISMA) flow diagram A total of 1,246 records were identified through database searches. After removing 316 duplicates, 930 records were screened. Of these, 124 full-text articles were assessed for eligibility. Ultimately, 37 studies were included in the review, while 87 articles were excluded because they involved non-recreational or elite populations or lacked validity and reliability data.

Systematic review of literature: A Preferred Reporting Items for Systematic Reviews and Meta-Analyses (PRISMA) 2020-guided systematic review, prospectively registered with PROSPERO (CRD420251023725) [16], was conducted (see Figure 1). Electronic searches were performed in PubMed, Scopus, Web of Science, Science Direct, Taylor & Francis, and Google Scholar for studies published between 2000 and May 2024. Search terms included combinations of pre-participation screening, recreational athletes, cardiovascular assessment, musculoskeletal evaluation, neuromuscular assessment, and fitness testing. After deduplication and multi-stage screening, 37 studies met the eligibility criteria. Extracted PPE components were categorized into five domains: medical history, musculoskeletal screening, cardiovascular assessment, neuromuscular control, and general fitness. This synthesis yielded 65 preliminary items, forming the Phase I item bank.

Item pool generation: Based on the review findings, a first-order list of items was developed to reflect major health and readiness domains. Items were worded in simple, non-technical language to ensure feasibility in recreational settings, where evaluators may have varying levels of medical training [17].

Expert review and refinement: The initial item pool was circulated among experts for structured evaluation. Experts assessed items for relevance to recreational athletes, practical applicability, clarity, and redundancy. Feedback was collected using standardized rating sheets and qualitative comments. Redundant or unclear items were removed or reworded, and missing areas, particularly cardiovascular risk and musculoskeletal function, were addressed. Consensus was achieved through iterative review, resulting in a refined draft PPE tool suitable for validation.

Outcomes of Phase I: Phase I resulted in the identification of core PPE domains specific to recreational athletes, the generation and refinement of an evidence-based item pool, and the development of a draft PPE tool endorsed by expert consensus. This draft instrument formed the basis for Phase II validation.

Phase II: PPE development and validity study (qualitative validation design)

Research Design

Phase II focused on establishing face validity, content validity, and the feasibility of the draft PPE tool. Validation relied on expert judgment and participant feedback rather than hypothesis testing. The phase comprised three sequential steps: face validity assessment, content validity assessment using Lawshe’s CVR, and pilot testing [10,11].

Participants

Experts: A minimum of 20 experts were purposively recruited, including orthopaedicians, physiotherapists, physicians, and sports medicine specialists. Inclusion was based on willingness to provide a professional evaluation. Larger expert panels are recommended to enhance the stability of content validity estimates. The sample size for experts was set at a minimum of 20, consistent with methodological literature. Armstrong et al. (2005) and Zamanzadeh et al. (2015) have both highlighted that larger expert panels provide more reliable consensus in content validity calculations, particularly when using Lawshe’s CVR formula. A smaller panel might compromise the stability of CVR values, whereas including 20 or more experts ensured that the critical cut-off value of 0.49 was applicable and meaningful [14,15,17,18].

Recreational athletes: For pilot testing, 30 recreational athletes aged 18-40 years were recruited using convenience sampling. Participants were engaged in high-intensity sports and met predefined eligibility criteria. A sample size of 30 is considered sufficient for pilot testing new instruments [19].

Sub-phases

Face validity: Experts evaluated whether the PPE tool appeared to measure athlete readiness appropriately and whether items were clear, relevant, and suitable for recreational athletes. Feedback regarding wording, sequencing, and format was incorporated to enhance acceptability and clarity.

Content validity: Content validity was assessed using Lawshe’s CVR [14]. Experts rated each item as: Essential, Useful but not essential, and Not necessary.

CVR was calculated as: $\mathrm{CVR} = \frac{2n_e - N}{N}$

where n_e_ represents the number of experts rating an item as essential, and N denotes the total number of experts. According to Lawshe’s criteria, items with a CVR value of 0.49 or above were retained. Items that failed to meet this threshold were either revised for clarity or removed from the tool. This process ensured that only those items deemed essential by a consensus of experts were included. The results of the CVR calculations were documented in a tabular format, which indicated the CVR values of each item along with decisions on whether they were retained, revised, or eliminated. Through this systematic process, the content validity of the PPE tool was established, ensuring that the items collectively reflected the essential domains identified in Phase I and were endorsed by professional judgment.

Pilot study: The refined PPE tool was pilot-tested on 30 recreational athletes under conditions simulating real-world use. Feedback was obtained regarding clarity, completion time, and comfort. Observations related to administration feasibility were also recorded. Based on feedback, minor revisions were made to improve usability and clarity.

Outcomes of Phase II: Phase II resulted in a final, content-validated PPE tool demonstrating: strong professional endorsement, conceptual clarity, and practical feasibility for recreational athletes.

Sampling Strategy

Experts: Experts were recruited using purposive sampling, appropriate for content validation studies requiring subject-matter expertise. The multidisciplinary panel ensured comprehensive coverage of medical, musculoskeletal, and functional domains.

Athletes: Athletes were recruited using convenience sampling, a pragmatic approach suitable for pilot studies. Inclusion criteria ensured representation of typical recreational high-intensity athletes.

Sample size justification: A panel of at least 20 experts was included to ensure stable CVR calculations and reliable consensus [14,17]. A sample of 30 athletes was considered adequate for identifying feasibility and clarity issues during pilot testing [18].

Data analysis

Data were analysed using IBM SPSS Statistics for Windows, Version 22 (Released 2013; IBM Corp., Armonk, New York, United States). Descriptive statistics were used to summarize participant demographics and feasibility outcomes. Content validity was quantified using Lawshe’s CVR and I-CVI. No inferential statistical testing was performed, as the study was methodological and qualitative in nature. This systematic approach ensured transparency, rigor, and reproducibility in the validation process.

## Results

Phase I: establishment of assessment domains and item development

The first step involved the identification of primary areas to assess from the literature review and analysis of numerous previously tested assessment frameworks. Over one dimension was being assessed, for example, history, musculoskeletal, cardiovascular, neuromuscular control, and overall fitness markers. Selection of these domains was made on the basis that a total measurement of the participants would be needed in a manner such that potential threats to health and performance limitations should be ruled out prior to commencing sports activities. Once the domains were identified, the initial bank of assessment items was developed. Expert consultation was an essential component of item finalization in terms of their relevance, clinical utility, and clarity expectations. The opinions of orthopaedics specialists, physiotherapists, sports medicine specialists, and general doctors were integrated, drawing upon evidence-based expertise and experience. The multidisciplinary approach allowed to integrate numerous perspectives and ultimately developed a series of initial evaluation measures tailored to leisure-time athletes.

Phase II: tool development, face validity, and pilot testing

The second step consisted of the construction of the PPE tool and testing it for face and content validity. Content validity was sustained through the pairing of assessment items with theoretical and practical requirements to ensure all significant health and performance variables are addressed comprehensively. All items were rated by expert reviewers for a number of characteristics in terms of relevance, comprehensibility, and necessity, and their suggestions guided systematic revision. Those items either unclear or redundant were changed or removed to enhance the effectiveness of the tool. Face validity test involved assessment of the extent to which the scale was communicating the intended objectives to the subjects and evaluators. This was achieved through expert reviews and observation of the participants during pilot testing. The parameters examined during pilot testing were instructions clarity, administrability, time sufficiency, and overall awareness of the scoring scheme. The good acceptance scores issued by participants indicated that the tool was well-designed and simple to administer. Additionally, pilot testing indicated some minimal areas that would need to be altered. These updates made the tool more user-friendly and versatile to fit any recreational setting. The result of this phase was a robust tool that was well-crafted and could be utilized operationally. Table 1 shows the demographic and training characteristics of participants (recreational athletes).

**Table 1:** Demographic and Training Characteristics of Participants (n = 30)

Variable	Category	N (%)
Total Participants = 30		
Age (years)	Mean ± SD	26.8 ± 5.4
Gender	Male	19 (63)
	Female	11 (37)
Type of Sport	Football	8 (27)
	Badminton	7 (23)
	Basketball	5 (17)
	Tennis	5 (17)
	Running	5 (17)
Training Frequency	2 sessions/week	5 (17)
	3-4 sessions/week	16 (53)
	≥5 sessions/week	9 (30)
Duration of Training	1-3 years	7 (23)
	3-6 years	14 (47)
	>6 years	9 (30)

Thirty recreational athletes participated in the study, with a mean age of 26.8 ± 5.4 years. There were 19 males (63%) of the participants. Football was the most commonly reported sport, n = 8 (27%), followed by badminton, n = 7 (23%). The majority of athletes trained three to four times per week, n = 16 (53%), and nearly half had been participating in high-intensity sports for three to six years, n = 14 (47%).

Expert panel demographics

The panel of experts constituted a broad spectrum of members with cross-specialization in various fields of medicine and allied health, thereby guaranteeing an extensive appraisal of the PPE tool. The input of specialists in non-clinical fields was a sought-after feature in an effort to achieve multidisciplinary and balanced analysis of the tool's content and structure, as well as clinical usability. Six orthopaedic experts among the 24 specialists provided invaluable feedback concerning musculoskeletal health and injury screening. Their experience was particularly valuable in refining products involving joint integrity, flexibility, chronic injury, and rehabilitation needs, all of which are focal to pre-participation examination of recreational athletes. Five physiotherapists were also instrumental in assessing physical performance and recovery factors, which involved components that were concerned with functional mobility, neuromuscular control, and general physical conditioning. Their function was pivotal when it came to determining that the tool was valid in quantifying indicators of motor coordination, strength, and endurance, which are highly pertinent in forecasting readiness for sports participation. Additionally, among five expert opinions of sports medicine experts, there was a performance vision, where the evaluation of sport-related areas involved cardiovascular activity, exercise intolerance, and work-related hazards. Their involvement improved the validity of the assessment process in that it centers on the reality that the tool aligns with evidence-based screening methods of non-elite athletes. The panel also included four doctors whose efforts were crucial in ensuring the support of the medical screening aspect of the PPE tool. The fields they evaluated stretched, among other areas, to individual and family history of medical problems, metabolic disorders and chronic illnesses, allergies, and medication histories. Such a comprehensive evaluation meant that the instrument measured wider concerns in health, yet without losing its practice-oriented character on sport participation and the recreational level in sports. The panel possessed varied experiences, and therefore, the input was extensive during the face validity stage and the content validity approach. Elaborative comments were provided regarding the clarity, pertinence, and completeness of every item, which enhanced the assessment framework. The clinical experience of panel members averaged at 12.8 ± 4.2 years, which reflects a fairly high level of professional interaction with the aspects of health management and functional evaluation practice in sports. The variety of people who participated in the development of the tool ensured the inclusion of clinical, rehabilitative, and performance-based aspects, which contributed to the validity of the tool in general. Table 2 shows the composition of the expert panel that participated in the validation of the PPE tool regarding their qualifications, specializations, and years of experience. The range of professionals brought about in-depth feedback in face and content validity tests.

**Table 2:** Expert Panel Characteristics

Variable	Category	n (%)
Total Experts		20
Specialty	Orthopaedicians	6 (30)
	Physiotherapists	5 (25)
	Sports medicine experts	5 (25)
	Physicians	4 (20)
Mean Clinical Experience	Years ± SD	12.8 ± 4.2
Role in Validation	Face validity assessment	20 (100)
	Content validity rating	20 (100)
	Item refinement	18 (90)

Phase I results: systematic review and item generation

Phase I aligns with the systematic analysis phase. A formal review of prevailing PPE frameworks, facilitated through expert consultation, was undertaken to determine relevant domains and constructs for the novel tool. A systematic analysis guaranteed that the new PPE instrument addressed the specific demands of recreational athletes. The results of Phase I, which were to determine essential domains of the PPE tool and create a structured item pool, are reported in this section. A synthesis of systematic reviews of existing assessment frameworks and expert opinion was utilized to develop an evidence-based and clinically practical framework for recreational participants in high-intensity sporting activities. The study was concerned with crafting a universal framework that would determine the risk of health-related injury, assess physical readiness, and predict the risk of injury. Integration of different clinical perceptions guaranteed that the instrument would enable all the significant ingredients leading to safe participation in leisure activities.

Identification of Key Domains

Review of already published sources and evaluation scales saw five most significant areas that are critical to physical preparedness and prevention of injury in sports players at an amateur level. These areas ensure that the evaluation covers medical, functional, and performance factors appropriately:

Medical history: The medical history field identifies conditions that can affect sports activities. It examines previous admissions, present treatments, existing conditions, allergies, and medication prescribed. The addition of these parameters will enable identifying existing conditions at the beginning and the likelihood of impacting exercise tolerance and preventing adverse effects that could arise during high-intensity physical activities.

Previous musculoskeletal injuries: Past injuries play a significant role in functional capacity and susceptibility to subsequent injury. This area evaluates past injuries of the bones, joints, muscles, and ligaments with regard to chronic pain, joint instability, and rehabilitation history. Pattern recognition of the same will facilitate modularization of training loads as well as the need for certain conditioning prior to high-intensity involvement in sports.

Cardiovascular health: The incorporation of high-intensity activities places significant stress on the cardiovascular system. The domain assesses cardiovascular well-being with such factors as blood pressure, heart rate variability, exercise tolerance, and the existence of family cardiac issues. Pre-screening for potential cardiovascular dangers assists in revealing a predisposed condition and susceptibility to the ill effects of physical exercises, and safe involvement in the physical activity.

Neuromuscular control: Neuromuscular coordination is a crucial component of a person, as it facilitates balance, postural stability, agility, and motor economy. Deficiencies in this component can be linked with the inability to perform at an optimal level and be susceptible to injuries. The parameters that are assessed are coordination during the execution of dynamic movements, proprioceptive regulation, and balance economy. Identification of deficits in these categories prevents falls and injuries, especially in cases of high-intensity, high-pressure, rapid maneuvers.

General fitness parameters: Functional capacity for fitness is the ability of the person to sustain a safe physical exercise. Tests employed in this field measure endurance, flexibility, muscular strength, body composition, and aerobic levels. Measurement of these baseline measures will determine physical limitations that are intrinsic as well as suggest appropriate conditioning before the individual engages in recreational sports. Table 3 shows the five main domains of the PPE tool and their focus areas and main constructs or indicators. It highlights priority areas such as medical history, musculoskeletal injuries, cardiovascular fitness, neuromuscular control, and overall fitness to present a comprehensive evaluation of the player.

**Table 3:** Identified Domains and Key Constructs

Domain	Primary Focus	Key Constructs/Indicators
Medical History	Identifying health-related risks	Chronic illness, surgeries, medications, allergies, and hospitalization history
Musculoskeletal Injuries	Understanding injury influence	Joint instability, flexibility, pain history, and rehabilitation records
Cardiovascular Health	Screening exertion-related risks	Blood pressure, heart rate variability, exercise tolerance, cardiac risk profile
Neuromuscular Control	Improving motor performance	Balance, coordination, proprioception, and reaction timing
General Fitness	Evaluating physical capacity	Endurance, body composition, strength, flexibility, and aerobic performance

Systematic Review Findings

The methodical evaluation of the available pre-participation assessment tools showed considerable shortcomings in the currently existing models with respect to recreational players. The design of the majority of assessment models is related to competitive or professional athletes only, which restricts the possibility of applying them to non-professional populations. Several gaps were identified: (1) Lack of adequate musculoskeletal screening: most of the existing tools lacked movement tests, joint stability, and balance, all of which are necessary in recreation; (2) Poor cardiovascular assessment: deliberate cardiovascular screening tests were covered in a few frameworks, even though it is important during high-intensity engagement; (3) Underrepresentation of neuromuscular evaluation: neuromuscular tools were not used very often and did not assess motor coordination, proprioceptive control, and agility, which are essential in injury prevention; and (4) Unavailability of recreational athlete-specific guidelines: guidelines were more about elite athletes; thus, there is a need to establish a focused framework of recommendations applicable to amateur sports participants. By filling these gaps, the proposed PPE tool will combine in one systematic format medical, functional, and performance-related indicators to enhance the quality of screening overall. This systematic analysis validated that musculoskeletal, cardiovascular, and neuromuscular screenings were underrepresented in current instruments. A combination of literature results and consensus opinion warranted the inclusion of these areas on the PPE tool.

Item Pool Development

Hence, the results of the systematic analysis in Phase I laid out the empirical base for item generation. The development of the preliminary item pool was done by integrating the results of a systematic review with the use of expert integrity to make it accurate and clinically meaningful. As consultations with specialists continue to evolve, semi-structured consultations were conducted with a multidisciplinary panel of 20 experts, including orthopaedicians, physiotherapists, sports medicine specialists, and physicians, to define the items and refine the set.

A total of 65 initial items were generated and distributed across five identified domains: Medical History (15 items), Musculoskeletal Screening (18 items), Cardiovascular Assessment (12 items), Neuromuscular Control (10 items), and General Fitness Indicators (10 items).

Experts judged the relevance, the clearness, and the feasibility of the assessment of each item. Duplicates and non-clinically relevant items were removed to leave only a substantial but not overwhelming body of items. The systematic information in a structured format facilitated a systematic assessment of health-related, functional, and performance risks in recreational athletes.

Expert Consensus Outcomes

A multidisciplinary panel was used to ensure that a clinically sound screening tool was developed. There was consensus on the core elements such as cardiovascular risk screening, dynamic balance testing, flexibility examination, and anthropometric analysis. Utilizing functional capacity measures like agility drills and endurance tests to enhance predictive accuracy for injury risk was also emphasized in recommendations. This cooperative approach resulted in a smoother and more evidence-based initial item pool upon which other validity testing and reliability analysis could be conducted at subsequent stages.

Phase II results: development, validity testing, and pilot assessment

Phase II was the validation testing of the PPE instrument. The phase entailed three steps: face validity, content validity, and pilot testing. These were carried out in order to ascertain that the items on the instrument were clinically relevant, statistically adequate, and practically tolerable among recreational athletes. Phase II was the purpose to methodically refine, validate, and practically pilot the PPE test designed for screening recreational athletes of high-level sport participants. This phase was undertaken for the sake of furnishing the clarity, clinical relevance, and statistical adequacy of the measuring instrument and the feasibility of the sample evaluation tool in the field. This process was divided into three key steps that involve a face validity test, a content validity test, and a pilot administration. All these steps resulted in the production of brief, dependable, and scientifically valid instruments usable in readiness and safety assessment of recreational athletes.

Face Validity Findings

Face validity was carried out by a panel of 20 experts with varying years of experience in field disciplines as varied as orthopaedics, physiotherapy, sports medicine, and general medicine. The experts reviewed the original list of 65 items to determine their clarity, intelligibility, and ease of targeting the purpose of pre-participation screening. These were evaluated based on how sensitive they were in detecting the risk of health issues, functional issues, and vulnerability to injury among the recreational players. The assessment revealed that 56 of the original items had been highly understood and were suitable to be used directly. Six items also required minor changes in formulations, e.g., clarifying technical writing and altering question ordering, as well as in clarity of context, which will allow for easier administration. Three items were eliminated after expert consensus that these did not make important contributions to the overall objectives or duplicated other items already measured in the assessment. This refinement contributed to a proper balance of the footprint on all main domains, such as medical background, skeletal stability, cardiovascular preparedness, muscular control, and overall fitness. Expert consensus established face validity by ascertaining that the items that were retained were clear, relevant, and consistent with the goals of pre-participation screening. Table 4 presents the layout of items accepted by the experts in face validity testing. It indicates the number of questions and the percentages of the questions that are retained, changed, and excluded from the initial pool of 65 questions, indicating that the majority of the questions were graded as clear and clinically accepted.

**Table 4:** Expert Ratings for Face Validity

Category	Number of Items (N)	Percentage (%)
Retained	56	86
Modified	6	9
Eliminated	3	5
Total	65	100

Content Validity Results (CVR Analysis)

The content validity was determined to evaluate the relevance and the requirement of each item statistically based on the CVR. Each of the 20 experts rated on a separate scale the items as purely essential, useful but not essential, or not necessary. The CVR was computed according to these ratings, but with a low threshold of 0.49 for item inclusion. Utilization of Lawshe's CVR yielded a statistical rationale where items with CVR ≥ 0.49 were kept, items slightly lower were revised, and items significantly lower than that were dropped. This preserved the inclusion of items using both expert judgment and statistical standards. The analysis showed 52 items with their CVR equal to or higher than the acceptance threshold, and did not make any changes. Seven items received scores just below the standard but were updated as per the suggestions of experts to improve their clarity, structure, and contextual relevance. The six items did not pass both clinical and statistical significance, resulting in items being dropped in the final structure. Table 5 shows the results of CVR analysis, the number of items that were retained, revised, and eliminated after being rated by experts. As it turned out, the largest proportion of items (over 70%) showed results that exceeded the set bar of CVR ≥ 0.49 and were kept in the final structure.

**Table 5:** Item-Wise Content Validity Ratio (CVR)

Outcome	Number of Items (N)	Percentage (%)
Retained (CVR ≥ 0.49)	52	80
Revised	7	11
Eliminated	6	9
Total	65	100

I-CVI

In addition to the CVR, the I-CVI was calculated to assess the relevance and clarity of each item. All 20 experts rated every item on a 4-point relevance scale. The I-CVI for each item was computed by dividing the number of experts who assigned a rating of either 3 (“relevant”) or 4 (“highly relevant”) by the total number of experts. An I-CVI value of ≥ 0.78 was considered acceptable for inclusion.

The analysis showed that 51 items achieved an I-CVI of ≥ 0.78 and were retained without modification. Eight items had I-CVI scores slightly below the acceptable range and were revised for clarity based on expert feedback. Six items demonstrated low I-CVI scores and were therefore removed, as they did not meet the minimum criteria for relevance and did not contribute meaningfully to the overall construct. These findings supported the CVR results and further strengthened the content validity of the PPE tool. Table 6 presents the distribution of items based on their I-CVI values.

**Table 6:** Item-Level Content Validity Index (I-CVI) Summary

Outcome	Number of Items (N)	Percentage (%)
Retained (I-CVI ≥ 0.78)	51	78
Revised	8	12
Eliminated	6	10
Total	65	100

Pilot Testing Outcomes

Pilot testing was done with a group of 30 recreational players, who volunteered to participate based on different high-intensity activities, which included badminton, football, tennis, basketball, and running. The main aim of this phase was to test the practical usefulness, understandability, and overall viability of the PPE tool within a more controlled environment of a sports clinic. The participants who completed the tool confirmed that the tool was easily understood and well-received. When asked whether the instructions were easy and clear, 27 (90%) of participants said yes, with 26 (87%) of participants saying that there was no difficulty in completing the assessment within the given time. The mean time spent on the evaluation process was about 35-45 minutes; many respondents found it comfortable and not time-consuming. Evaluators also described unhindered administration as a result of the standardized structure that reduced inconsistency in related procedures and simplified scoring.

Minor changes based on the collected feedback were conducted, such as a refined wording in particular areas and rearranging of item sequence to provide logical progression and simplification of technical vocabulary where it was possible. The changes facilitated the ability to intuitively interpret the instrument by both participants and assessors, and maintain the clinical potential of the instrument. Pilot testing validated the results of face and content validity since the athletes reported high rates of clarity, usability, and feasibility, thus affirming the validity of the PPE tool in real-world contexts. Table 7 shows the responses of the participants and evaluators made during the pilot test stage in relation to the usability, understandability, and practicality of the PPE tool. High positive response rates suggest test simplicity, time effectiveness, and gainful acceptance by recreational players.

**Table 7:** Pilot Testing Feedback

Feedback Parameter	Positive Responses (n)	Percentage (%)
Clarity of Instructions	27	90
Ease of Administration	26	87
Time Feasibility	25	83
Overall Comprehensibility	27	90
Suggested Modifications	3	10

The results of the pilot testing showed that the PPE tool had high scores referencing usability, acceptance among participants, and feasible operations. The changes made in this phase enhanced the usability and understandability of the tool even further.

Finalization of the PPE tool

Following professional editing and pilot testing, the last PPE instrument included 59 stable items that were spaced between five important domains. These were 14 items to provide medical history, 16 items to evaluate musculoskeletal, 11 items of cardiovascular evaluation, nine items of neuromuscular control, and nine items of general fitness. The final design gave wide coverage of screening, but it was concise and easy to handle, allowing efficient implementation without the imposition of losses to clinical completeness.

## Discussion

Principal findings

The present study developed and established the content validity of a novel PPE instrument specifically tailored for recreational athletes engaged in high-intensity sports. The findings demonstrate excellent face validity and strong content validity, with I-CVI values exceeding established methodological thresholds. In accordance with contemporary measurement theory, robust content validity provides a critical foundation for subsequent psychometric evaluation, including reliability and construct validity testing [11].

Validity outcomes

Face Validity

Face validity was assessed by a multidisciplinary panel of 20 experts drawn from orthopaedics, physiotherapy, sports medicine, and general medicine. Expert review resulted in the revision of approximately 9% of the original 65 items to enhance clarity and comprehensibility, while 30% of the items were eliminated due to redundancy or limited relevance. The remaining 86% of items were retained, reflecting strong professional endorsement. The high proportion of retained items indicates that the tool was perceived as clinically relevant, clearly worded, and aligned with the objectives of pre-participation screening. Importantly, expert feedback also emphasized the usability of the instrument for both clinicians and recreational athletes, supporting its practical applicability.

Content Validity

Content validity was evaluated using Lawshe’s CVR method. Of the 52 items subjected to CVR analysis, 80% achieved a CVR ≥ 0.49, meeting the recommended threshold for panels of 20 or more experts. Seven items (11%) were revised to improve contextual relevance, while six items (9%) were removed. These findings indicate that the PPE tool effectively captures essential domains of athlete readiness, including medical history, musculoskeletal health, cardiovascular preparedness, neuromuscular control, and general fitness, without unnecessary redundancy. High CVR values confirm strong expert consensus regarding item essentiality, thereby reinforcing the scientific rigor of the instrument.

The validity outcomes are consistent with prior PPE development studies that emphasize expert-driven content validation as a cornerstone of screening tool development. The proportion of items exceeding the CVR threshold in this study aligns closely with established methodological standards [20,21].

Pilot Testing and Practical Validity

Pilot testing conducted among 30 recreational athletes demonstrated favourable practical validity. The majority of participants reported a clear understanding of instructions (90%), ease of administration (87%), and acceptable time feasibility (83%). Completion time ranged between 35 and 45 minutes, which was considered reasonable for a comprehensive pre-participation assessment. Minor refinements, including rephrasing technical terminology and improving item sequencing, were implemented based on participant feedback. These findings confirm that the PPE instrument is not only valid in content but also feasible and acceptable for real-world use in community and clinical sports settings [19].

Final Tool Composition

The final validated PPE tool consists of 59 items distributed across five domains: Medical history (14 items), musculoskeletal health (16 items), cardiovascular fitness (11 items), neuromuscular control (nine items), and general fitness (nine items). This balanced domain representation ensures comprehensive coverage of physiological and functional parameters relevant to high-intensity recreational sports participation.

Comparison with existing PPE frameworks

Traditional PPE tools, particularly those used in scholastic and elite sport, are largely consensus-based and infrequently subjected to formal validity testing [7,8]. Additionally, these tools often overlook contextual factors unique to recreational athletes, such as irregular training patterns and limited medical oversight. The present PPE instrument addresses these limitations by specifically targeting recreational athletes, incorporating training load and recovery considerations, recognized as critical injury predictors [22], and employing COnsensus-based Standards for the selection of health Measurement INstruments (COSMIN)-aligned validation methodology, which remains underutilized in PPE research.

Furthermore, the inclusion of neuromuscular and general fitness domains extends earlier PPE models that focused primarily on performance and return-to-play decisions, aligning instead with contemporary preventive screening paradigms [22].

Clinical and practical implications

A PPE instrument with strong content validity may facilitate early identification of individuals requiring further medical evaluation, particularly for cardiovascular and musculoskeletal risks. Given the low incidence but potentially catastrophic outcomes of sudden cardiac events in sport, even modest improvements in screening relevance may yield meaningful public health benefits. The clarity, feasibility, and contextual relevance of the instrument support its implementation in community gyms, recreational leagues, and fitness centres, addressing recognized limitations of existing PPE practices [3,22].

Methodological strengths

Key strengths of this study include a theory-driven construct definition, inclusion of both subject-matter experts and end users, use of quantitative (CVR) and qualitative validation methods, and transparent decision rules for item revision and retention. These elements align with best-practice recommendations for content validity assessment and enhance the methodological credibility of the findings [11,13].

Limitations

Several limitations warrant consideration. Although the expert panel size met methodological recommendations, generalizability may be limited. Additionally, content validity alone does not establish reliability or predictive accuracy. Cross-cultural applicability was not assessed, which is important given the global growth of recreational high-intensity sports. As the study was conducted at a single institution with a relatively homogeneous sample, the generalizability of findings may be limited. Validation across multiple centres, geographic regions, and culturally diverse recreational athlete populations is required. Although administration time ranged from 35 to 45 minutes, the PPE tool was designed as a comprehensive screening instrument rather than a rapid field checklist. This duration is comparable to detailed PPE protocols used in clinical settings. Future research may explore the development of a shortened version to enhance feasibility in time-restricted environments. As this study focused on early-phase instrument development, findings are limited to face validity, content validity, and feasibility. External validity, reliability, and predictive accuracy were not assessed and represent essential next steps. Future studies should evaluate test-retest reliability, construct validity, and injury-prediction capability in larger, multi-centre, and culturally diverse recreational athlete populations.

Future directions

Future research should assess test-retest reliability and internal consistency, establish construct validity using known-groups and hypothesis-testing approaches, evaluate predictive validity for injury and adverse events through longitudinal studies, and conduct cross-cultural adaptation and re-validation. Such investigations are essential before widespread clinical and community implementation.

## Conclusions

The present study developed a PPE instrument specifically designed for recreational athletes engaged in high-intensity sports and established its face validity, content validity, and feasibility. Using a rigorous, theory-driven methodology that incorporated systematic literature review, multidisciplinary expert consensus, and pilot testing, the instrument demonstrated strong professional endorsement and practical acceptability. While the PPE tool shows excellent content validity across medical, musculoskeletal, cardiovascular, neuromuscular, and general fitness domains, further psychometric evaluation is required. Future studies should assess test-retest reliability, internal consistency, construct validity, and predictive validity in larger and more diverse populations before widespread implementation. Nevertheless, this study provides a robust foundation for recreational-athlete-specific screening and addresses important gaps in existing PPE frameworks.
